# Therapeutic Effects of Cycling on Disability, Mobility, and Quality of Life in Patients Post Stroke

**Published:** 2019-02

**Authors:** Ardalan SHARIAT, Noureddin Nakhostin ANSARI, Joshua A. CLELAND, Azadeh HAKAKZADEH, Ramin KORDI, Mehdi KARGARFARD

**Affiliations:** 1. Sports Medicine Research Center, Neuroscience Institute, Tehran University of Medical Sciences, Tehran, Iran; 2. Department of Physiotherapy, School of Rehabilitation, Tehran University of Medical Sciences, Tehran, Iran; 3. Franklin Pierce University, Manchester, New Hampshire, USA; 4. Department of Exercise Physiology, Faculty of Sports Sciences, University of Isfahan, Isfahan, Iran

## Dear Editor-in-Chief

The prevalence of some diseases, such as stroke, is increasing in developing countries. The reason for this increase is not fully understood but one potential cause is the increasing life expectancy. “Of 5.7 million patients who had a stroke and died in 2005, 87% were from low- and middle-income countries” (1). In these countries, stroke is considered a major disabling health problem and because of the high cost of drugs/treatments, the majority of people are not able to access these resources (1).

Researchers still do not fully understand the causes of stroke among the young population or why its prevalence is increasing (2). In most cases, cardiac embolism and arterial dissection, and less frequently, genetic causes can have been associated. Therefore, suggesting a home-based solution with low cost may potentially be useful for this population of patients especially in developing countries, where there has not been enough research with a focus on practical solutions for treatment/rehabilitation. The first aim of this home-based solution was to reduce the disability and improve the mobility by improving recovery of volitional movement or replacing lost volitional movement which will overall improve the quality of life.

In rehabilitation of patients who have had a stroke, improving ambulation is one of the major goals. Gait training requires therapist assistance and also costly computer controlled devices as ortho-skletons. Therefore there is a need for the development of convenient and inexpensive rehabilitation strategies.

Cycling is one of the easiest ways to train the patients with disability and has been used in previous studies (3,4), as it is easy to control the intensity of exercise training and it is also safe. Furthermore, it can be adjusted to the complexity of the patient with starting as cycling in the lying position progressing to cycling in the seated and supine position in different level of disability, on land or in water (5).

There exists a plethora of valuable research about the therapeutic effects of cycling on mobility, gait, disability, and quality of life among patients post stroke (4,6–8). However, it is still not clear if, different durations and intensities of cycling with or without support can have different effects on disability, mobility, gait, and quality of life among patients post stroke. In addition, it is still unclear if different protocols and intensities and durations should be used for different ages and genders. In addition, previous studies (8), cover a wide range of duration after stroke, such as 1 wk to 50 wk, but it is still not clear if they need different protocols of exercises. In addition, the benefits of using functional electrical stimulation with cycling need to be examined for different age groups, gender, intensity, duration, and history of stroke.

There is also an additional gap in previous studies as most performed a pre-test, one post-test after 4–6 wk and they’re follow up was complete after 3 months. It is not clear the exact time necessary to maximize improvement in mobility, disability and gait so longer-term studies are required.

As the prevalence of stroke continues to increase must identify practical and cost-effective methods for improving mobility and quality of life. Identifying these could result in substantial reduction in patients suffering as well as reduce socioeconomic burdens. We are hopeful that researchers and clinicians will continue to examine effective and cost-efficient strategies to assist with improving the overall quality of individuals post-stroke.
